# ‘It makes life so much easier’—experiences of users of the MicroGuide™ smartphone app for improving antibiotic prescribing behaviour in UK hospitals: an interview study

**DOI:** 10.1093/jacamr/dlab111

**Published:** 2021-08-12

**Authors:** Kieran S Hand, Bridget Clancy, Mike Allen, Amazigom Mayes, Yash Patel, Susan M Latter

**Affiliations:** 1 Faculty of Environmental and Life Sciences, University of Southampton, Southampton, UK; 2 Merck Sharp & Dohme Limited, Hertford Road, Hoddesdon, UK

## Abstract

**Objectives:**

To understand the impact on prescribing behaviour of an antimicrobial therapy guidelines smartphone app, in widespread use in hospitals in the UK.

**Methods:**

Twenty-eight doctors and five nurse prescribers from four purposively selected hospitals in the UK participated in behavioural theory-informed semi-structured interviews about their experiences of using the MicroGuide™ smartphone app. Data were analysed using a thematic content analysis.

**Results:**

Five themes emerged from the interview data: convenience and accessibility; validation of prescribing decisions; trust in app content; promotion of antimicrobial stewardship; and limitations and concerns. Participants appreciated the perceived convenience, accessibility and timesaving attributes of the app, potentially contributing to more prompt treatment of patients with time-critical illness. The interviewees also reported finding it reassuring to use the app to support decision-making and to validate existing knowledge. They trusted the app content authored by local experts and considered it to be evidence-based and up-to-date. This was believed to result in fewer telephone calls to the microbiology department for advice. Participants recognized the value of the app for supporting the goals of antimicrobial stewardship by promoting the responsible and proportionate use of antimicrobials. Finally, a number of limitations of the app were reported, including the risk of de-skilling trainees, cultural problems with using smartphones in clinical environments and software technical problems.

**Conclusions:**

The MicroGuide app was valued as a means of addressing an unmet need for updated, concise, trustworthy specialist information in an accessible format at the bedside to support safe and effective antimicrobial prescribing.

## Introduction

Infection is a common medical condition and a cross-speciality problem, typically managed by doctors without specialist microbiology or infectious diseases training and trainee doctors report a lack of knowledge and confidence with regard to antimicrobial prescribing.^1–3^ Antimicrobial resistance (AMR) varies according to pathogen epidemiology, geographical location and clinical setting, adding to prescribing complexity, with antimicrobial overuse and misuse commonplace, potentially exacerbating the problem of AMR.^4–6^

Clinical guidelines are a commonly used antimicrobial stewardship intervention to facilitate safe and effective antimicrobial prescribing by non-specialists, with adherence to guidelines associated with reduced antimicrobial prescribing and lower mortality.^7–10^ Conversely, prescribing *off-guideline* is associated with prescribing of broader spectrum antibiotics but not necessarily with better targeting of the likely pathogens.^11^^,^^12^ Surveys of doctors reveal a demand for improved accessibility of guidelines via smartphones.^13–16^ Emerging evidence demonstrates that providing antimicrobial prescribing guidelines and policies through the medium of a mobile device application can improve guideline adherence and policy compliance,^17^^,^^18^ enhancing physician knowledge and impacting antimicrobial prescribing behaviour.^19^^,^^20^

MicroGuide™ is a software application (app) for mobile electronic devices and web browsers, providing locally adaptable antimicrobial treatment guidelines and infection management advice for common infections, developed in response from feedback from trainee doctors. MicroGuide was co-designed and developed in 2011–12 by Horizon Strategic Partners (HSP) Limited and University Hospital Southampton in the UK and, from 2013, the app was licensed to other healthcare organizations in the UK and internationally.^21^^,^^22^ MicroGuide is currently licensed to 147 healthcare providers in primary and secondary care in 18 countries, including 91 of the 152 acute hospital Trusts in the NHS in England and 5 healthcare organizations in the United States (E. Halpin, HSP, personal communication). Each hospital has editorial independence to modify the app content to reflect local antimicrobial prescribing guidelines and to customize the app navigation architecture according to the preferences of local clinicians.^23^ To date, the app has been downloaded free of charge over 250 000 times by users in 173 countries worldwide.

Published evaluation of the impact of the MicroGuide app has thus far been confined to a questionnaire survey of app users in a single UK hospital, a descriptive evaluation of the app implementation process in a single US hospital and interviews with app users in a single hospital in Malawi.^23–25^ No multicentre research has been reported and no interviews with clinicians using the app in high-income health settings have been published. The research presented here was undertaken at four acute NHS hospitals in the UK using interviews to gain insight into the experience of doctors and nurse prescribers of using the MicroGuide app to access antimicrobial prescribing guidelines and to explore app user perceptions of the impact of the app on their prescribing behaviour.

## Methods

### Setting

Interviews took place at four NHS acute hospital Trusts across the south of England, including two university-affiliated teaching hospitals and two district general hospitals, with bed numbers ranging from 500 to 1200. App users were interviewed in person, individually, while at their place of work in a suitable private area.

### Inclusion and exclusion criteria

All healthcare professionals at the four selected hospitals, who had registered with the app software developers and downloaded the MicroGuide app to a personal mobile device, and were actively prescribing antimicrobials, were eligible for interview. Clinicians who had moved out of area or could not be contacted were excluded from the study. Doctors included physicians and surgeons and senior doctors (consultants) were defined as those who had completed foundation (2 years) and specialist (6 years) training. Nurse prescribers were qualified as independent non-medical prescribers.

### Sampling and recruitment

Participating institutions were selected from a group of 37 acute hospital Trusts in the UK that had been using the MicroGuide app for a minimum of 2 years. Hospitals were stratified by intensity of app use, as represented by new user registrations and app access events, according to data provided by the software developer. App user activity was adjusted for hospital activity (number of admissions) and number of doctors employed 1 year following launch of the app at each institution. Two hospital Trusts from the highest quartile and two from the lowest quartile of app user activity were identified within feasible travelling distance of the research team and were recruited to the study.

The software developer identified all MicroGuide app users in the four selected hospitals from user registration information and invited interested users to share their contact details and app activity data with the research team. The Research Nurse (B.C.) contacted consenting respondents to provide a participant information sheet and an invitation to attend an interview. In order to capture the experiences of medical and non-medical prescribers of different levels of seniority, some of whom were relatively high-frequency and some low-frequency app users, contacts were made sequentially with MicroGuide users purposively sampled according to a maximum variation sampling frame (Table 1).^26^ Users within each sampling frame were contacted sequentially in order of frequency of access to the app—highest frequency users were contacted first in the high-frequency sampling frames and lowest frequency users were contacted first in the low-frequency sampling frame.

**Table 1.** MicroGuide app user interview sampling framework

Prescriber group	High-frequency app use				Low-frequency app use			
	Trust A		Trust B		Trust C		Trust D	
	planned interviews	actual interviews	planned interviews	actual interviews	planned interviews	actual interviews	planned interviews	actual interviews
High-frequency user prescribers^a^								
trainee doctor	3–4	4	3–4	3	3–4	4	3–4	3
senior doctor (consultant/attending)	1–2	2	1–2	2	1–2	2	1–2	2
non-medical prescriber	0–1	1	0–1	1	0–1	1	0–1	1
Low-frequency user prescribers^b^								
trainee doctor	1–2	1	1–2	2	1–2	0	1–2	1
senior doctor (consultant/attending)	0–1	0	0–1	0	0–1	1	0–1	1
non-medical prescriber	0–1	1	0–1	0	0–1	0	0–1	0
Total	8	9	8	8	8	8	8	8

a High-frequency user prescribers: 13 or more app access events in the preceding 24 months.

b Low-frequency user prescribers: 1–12 app access events in the preceding 24 months.

### Semi-structured interview methodology

The study was approved by the Health Research Authority for England following ethical approval by the University of Southampton (Ref: 18/HRA/0256). Written informed consent was obtained prior to each interview, which took place between October 2018 and January 2019 at a convenient time before or after clinical work shifts or during meal breaks at the participant’s place of work. Interviewees were offered a £30 Amazon voucher to compensate for participating during their personal time. Interviews were all conducted by the same Research Nurse (B.C.), who is trained and experienced in research interviewing, and lasted 14–38 min (mean 28 min). Interviews were audio-recorded and subsequently anonymized, then transcribed verbatim for analysis.

The interview topic guide (Table S1, available as Supplementary data at *JAC-AMR* Online) followed a framework derived from the capability, opportunity, motivation model for behaviour change (COM-B), which conceptualizes how an individual modifies their behaviour to accommodate change.^27^ The COM-B model is derived from the Theoretical Domains Framework, which distils constructs from 33 behaviour change theories into domains to explain common influences on behaviour and this framework has been used to design interview topic guides to explore the factors influencing antimicrobial prescribing in healthcare settings.^28^^,^^29^ The topic guide was used in a semi-structured way using follow-up questions and prompts to probe areas of interest and allowing the interviewee to develop responses beyond the scope of the guide.^30^

### Analysis

Interview transcriptions were imported into NVivo 12 data handling software package (QSR International) and were analysed thematically according to the method described by Braun and Clarke.^31^ Transcripts were initially read and coded by the interviewer and an inductive process was used to develop a coding framework derived from the interview data. This initial framework was subsequently populated with coded interview extracts then reanalysed to identify major themes and sub-themes. The coding framework and interview extracts were reviewed by a second member of the research team, trained in qualitative interview technique and thematic analysis (K.S.H.), to validate identified themes and sub-themes and any disagreements were debated with the interviewer until a consensus was reached. The resulting themes were then reviewed and further refined by discussion at meetings attended by all members of the qualitative research team (B.C., K.S.H., S.M.L.). An inductive process was employed to avoid constraining the findings to fit the COM-B framework.

## Results

One hundred and fifty-six out of 7109 registered MicroGuide users across the four selected NHS acute hospital Trusts agreed to be contacted by the research team and 72 individuals were contacted. Of those approached, 27 did not respond or an interview could not be arranged, 10 had moved away from their employing hospital and 2 were not required after interview theme saturation had been achieved. Theme saturation was achieved after 33 interviews. Interviewees included 18 doctors in training, 10 senior doctors (consultants) and 5 non-medical prescribers (all nurses). The distribution of interviewees by professional group, seniority, frequency of app use and participating hospital site is presented in Table 1.

Interviewees in all four trusts reported a high level of awareness of MicroGuide amongst their colleagues. Several interviewees reported that it was used more in the medical specialities than surgical specialities and some staff believed it to be more amenable to younger staff—a view contested by some of the senior doctor interviewees. Five major themes were determined from analysis of the interview data. Details of each of the themes and sub-themes with corresponding illustrative quotes from interviewees are summarized in Table 2. Each of the five themes is described in more detail in the following sections.

**Table 2.** Results of thematic analysis of MicroGuide app user interview data

Major themes	Sub-themes	Relevant interview fragment supporting theme
1: Convenience and accessibility	Quick and easy to use	*‘You’d have to be pretty thick not to be able to follow it. I think so, it’s very user friendly.’* (nurse practitioner)
		*‘I think the positive thing is the time saving and we’re only talking…maybe it saves five minutes a patient but you use it for six patients in a shift and there’s half an hour. That’s the difference between seeing another patient and not. So I like it.’* (training grade doctor)
	Validates referral to specialists	*‘I think it’s frustrating for microbiologists if they get calls about quite easy questions that are clearly answered by MicroGuide.’* (specialist trainee doctor)
		*‘It’s like calling Microbiology without calling Microbiology.’* (specialist trainee doctor)
		*‘Maybe the time where I wouldn’t use it is in complicated patients who have maybe had antibiotics before. I think for almost any indication where someone has already had antibiotics and its failed, that’s the time when I’d probably talk to Microbiology directly rather than using the app.’* (training grade doctor)
	Supports ‘in-the-moment’ decision-making	*‘If there’s a question for example on a ward round where someone says, oh is that the right antibiotic given that we now think it’s not chest it’s more likely to be urine or something? Normally there’s at least three of us that pull out our phone and it will range between the junior juniors, the registrar and the consultant.’* (training grade doctor)
		*‘It’s quite easy to access on a ward round so it means that you’ve done the job that you’ve been asked to do instantly rather than waiting to the afternoon where you then have to maybe go through the guidelines or have a discussion with micro about it.’* (training grade doctor)
	Facilitates prompt initiation of treatment	*‘If you’ve got someone acutely unwell needing antibiotics quite urgently you are digging into their golden hour quite considerably by having to go and find a computer somewhere. If you can look at it at the bedside, a minute later you’ve written a prescription and you can hand it to the relevant person.’* (nurse practitioner)
2: Confidence to validate prescribing decisions	Support for decision-making outside area of expertise	*‘I don’t know everything and I can’t remember everything.’* (specialist trainee doctor)
		*‘I think right from a senior level on ward rounds we’re encouraged to use it and it’s used as a decision-making tool for choice and the fact that consultants use it makes you use it as well.’* (training grade doctor)
	Integration with existing clinical expertise	*‘Actually probably eight or nine times out of ten if I was prescribing an antibiotic I would probably look to make sure we’re adhering to the guidelines.’* (consultant/attending physician)
		*‘It means that if someone senior, for example, a consultant is considering a particular antibiotic choice you can then have a discussion because it’s then very easy to then very quickly say well this is what the guideline would be.’* (training grade doctor)
	Integration with specialist advice	*‘From an antimicrobial guardianship that’s important as well and also the microbiologists having knowledge of what the local flora and fauna are, so what is the issue around here, what are local urinary tract infections sensitive to compared to what they might be in Southampton or wherever. I think that’s useful.’* (consultant/attending physician)
3: Trust in app content	Use as a ‘pocket expert’	*‘I guess my job is to diagnose what infection they’ve got and then rely on the experts who have made MicroGuide to tell me what antibiotics are most likely to be helpful’* (specialist trainee doctor)
		*‘MicroGuide puts all the information in one place which can be useful. It does mean it’s a more wieldy document which you couldn’t put on to a credit card sized thing, but it does mean that all the information is in one place.’* (consultant/attending physician)
	Faith in app content	*‘The advantages are that it’s quite slick and easy to use and I suppose I trust it because it’s ratified by NHS organizations.’* (training grade doctor)
		*‘Everyone’s MicroGuide is unique to the local population that the microbiologists have been having an input into.’* (consultant/attending physician)
	Value as learning tool	*‘We almost use it as a sort of test. If we turn around and say, “What would you prescribe?” and if they say something, we say, “Is that in line with what our local protocol tells us? Why don’t we check on MicroGuide?” so it almost acts as a back-up to almost teach them—the junior doctor—and go from there.’* (consultant/attending physician)
		*‘For me its educating me about the different—I’m still young in prescribing—the different antibiotics for different indications, different infections.’* (nurse practitioner)
4: Promotion of stewardship	Value to organization	*‘I would say that in the last few years the benefit has been clearly a drop or reduction in the use of some of the more expensive broad-spectrum antibiotics, for example, tazobactam and piperacillin, vancomycin or even carbapenems, so that has certainly been something which the app has probably enabled.’* (consultant/attending physician)
	Role in antimicrobial stewardship	*‘Obviously it’s better for the patient because you are not throwing them the strongest antibiotics you think about so it’s going to reduce resistance isn’t it?’* (training grade doctor)
		*‘I think it should be mandatory within the NHS. If there is so much research going on about in particular in a time where we are trying to preserve the antibiotics that we have I think that people should be using something that works and this is all research driven and it’s been proven that these are the right doses that we should be using.’* (nurse practitioner)
5: Limitations and concerns	Over-reliance on guidelines	*‘People blindly follow it and they don’t apply context and they don’t apply the patient in front of them, they just do what MicroGuide advises’* (specialist trainee doctor)
		*‘I don’t even know who has put the information on. No, I don’t know what the origin of that is, I have no idea at all.’* (specialist trainee doctor)
		*‘I suppose if there was someone that relies on it 100% and it does then switch their brain off from thinking then maybe that’s not great for them but […] it will still be fine for the patient, it would still be fine for the hospital and it would still be fine for the wider population, they just wouldn’t be learning.’* (training grade doctor)
	Use of smartphones in clinical areas	*‘You have to have a phone to do your job and yet the NHS is not prepared to contribute to that. I think it would be dead handy if there were better opportunities to be able to charge your phone around the Trust.’* (consultant/attending physician)
		*‘Clearly consultants and junior doctors have mobile phones in their pockets and clearly look at them on a regular basis so for them it’s fine, but for say the nurses and therapists, that would be something which they wouldn’t necessarily engage with.’* (consultant/attending physician)
	Technical glitches	*‘I’ve had a few issues with it not working. There was a period of time where it didn’t work very well and it kept crashing. I think it’s better now I’ve got a newer phone.’* (training grade doctor)
		*‘It does take a minute or two to come up. Really that’s not a big issue except in our modern rushed world, it’s like come on, come on.’* (consultant/attending physician)

### Theme 1: Convenience and accessibility

MicroGuide was reportedly well integrated into routine workflow due to its accessibility at the point of care facilitating *in-the-moment* decision-making, whether at the bedside, during the ward round or in the office. Users found the app quick and easy to consult, saving valuable minutes over the course of a day. This was noted as particularly beneficial during an acute episode of illness when prompt treatment is paramount.

*‘It’s much, much easier. And that’s why I have used it just simply because it’s available, it’s there, it’s with you all the time. We like ease and we’re all very techy aren’t we, we all carry our phones in our pockets.’*

(nurse prescriber)

### Theme 2: Confidence to validate prescribing decisions

MicroGuide was perceived as providing information and guidance to support safer prescribing and to reassure the prescriber. Many clinicians believed it improved their prescribing by acting as a ‘pocket expert’ to enhance their confidence and autonomy and to prompt when experiencing ‘information overload’.

*‘I don’t care that I don’t know every dose of everything. I’d just rather make sure. I know where I can find the information, it takes me two minutes, I care that I’m safe. It saves brain space. I love it. It makes life so much easier.’*

(training grade doctor)

MicroGuide was appreciated by many trainee doctor prescribers and those working outside their area of expertise, such as on-call or night shifts or as locum medical staff, as it offered security and alleviated the worry of making an error.

*‘You tend to prescribe within your area so common things are common to you and you know the policy for that but it’s when you step outside of your normal practice, you’ve got a child who perhaps has a chest infection. OK, what is the up-to-date policy first line for a chest infection because I don’t deal with a chest infection every day.’*

(nurse prescriber)

There was also evidence to suggest that MicroGuide was empowering trainees to challenge decisions of senior staff. MicroGuide was frequently used in place of a telephone call to microbiology for straightforward queries, enabling the microbiology team to focus on more complex queries.

*‘[Before we got MicroGuide] you’d either end up hammering the microbiology service hotline or prescribing whatever your boss wanted, which might not necessarily be the right thing.’*

(training grade doctor)

### Theme 3: Trust in app content

Clinicians described the content of MicroGuide as accurate, specific, comprehensive and trustworthy. The facility of hospital Trusts to update the contents frequently was appreciated by clinicians, who relied on it for the latest up-to-date prescribing guidelines.

*‘I don’t think updates on prescribing are disseminated very well, but because I logged onto MicroGuide and it had changed, I then knew to change my prescribing.’*

(specialist trainee)

Interviewees were content to refer to prescribing guidance in the app and did not believe their role of examining and diagnosing the patient was diminished by reference to the specialist prescribing knowledge it provided. Furthermore, the value of MicroGuide as a teaching instrument or for personal learning was frequently mentioned.

*‘I think it improves my knowledge of what antibiotics should be used for certain conditions. Even if I use it again to just double check myself, I’ve still got it in my mind about what is the correct antibiotic to use. So I think it’s actually improved my knowledge.’*

(training grade doctor)

### Theme 4: Promotion of stewardship

There was a widespread belief that MicroGuide provided a convenient platform for consulting Trust-specific guidelines, which consequently ensured that clinicians were more likely to follow them. Prescribers concurred that adherence to the guidelines would lead to greater consistency of prescribing and more predictable demand for drugs across the Trust, with the additional potential for financial savings.

*‘It definitely enables you to make sure that you are prescribing antibiotics properly and making sure that you are not encouraging resistance.’*

(training grade doctor)

Several interviewees, however, reported that colleagues sometimes chose not to adhere to guidelines and suggested that improved access to guidelines via the app may make little difference to their behaviour.

*‘There are people who have been doing things for years and years and know how they like to do it and what works for them and they’re not going to change their practice. They will always be outliers some of them because you can’t make people do stuff.’*

(consultant/attending physician)

### Theme 5: Limitations and concerns

Over-reliance on MicroGuide and its influence on decision-making concerned a small number of prescribers of all grades who perceived the potential to ‘get by’ with limited understanding. It was also suggested that the app might encourage clinicians to focus too heavily on biomedical treatment options, failing to consider the complete patient and the context of the illness.

*‘I would hope that most clinicians use it in conjunction with the patient in front of them and the context of the patient and their medical history but there is always a concern as soon as something is written down that people just blindly follow it.’*

(training grade doctor)

Updating and other technical issues were frustrating for some, blamed on the limited memory of older smartphones or inadequate Wi-Fi service around the hospital.

## Discussion

Testimony from experienced and trainee medical and non-medical prescribers presented here illustrates the challenges posed by information overload in contemporary healthcare and the hurried pace of modern hospital clinical practice. The findings of this research add to the growing body of evidence demonstrating the demand for trusted, credible information sources to support sound clinical decision-making that are reliably and readily accessible at the point of prescribing. Participants were candid in their acknowledgement of the limitations of individual practitioners to retain sufficient up-to-date knowledge of the appropriate use of antimicrobial agents and their responsibility to seek specialist information from resources such as MicroGuide and infection experts when necessary to safeguard patients.

Our findings are consistent with those of reports published by other hospitals using MicroGuide. Over 90% of 146 survey respondents at University College Hospital (UCH) in London agreed or strongly agreed that the app was the best way to access the hospital guidelines and this sentiment is reflected in the convenience and accessibility theme reported here.^24^ The local relevance of guideline content was described by doctors interviewed at a large urban Hospital in Malawi as crucial, particularly with regard to antibiotic availability and pathogen epidemiology.^25^ The antimicrobial stewardship theme was also observed in Malawi, where trainee doctors perceived MicroGuide to be a new resource that would guide them to narrow-spectrum antibiotics. Reflections by interviewees that telephone calls to the microbiology department were perceived to be less frequent since the introduction of MicroGuide resonates with survey findings from UCH that advice was sought from microbiology specialists less frequently following app launch, potentially freeing up specialists to deal with complex cases.

Concerns expressed by interviewees about a culture of disapproval of smartphone use in hospital ward settings is a limitation of apps reported previously^32^^,^^33^ although this contrasted with findings of the survey of doctors using MicroGuide at UCH, potentially reflecting a perception of greater tolerance of smartphone use by medical staff in contrast to nurses and therapists. The risk of medical de-skilling posed by guideline resources such as MicroGuide is another legitimate concern highlighted by some respondents, although at least one randomized study indicates that app use may be associated with an improvement of knowledge of antimicrobials.^34^ Limited available evidence suggests that smartphone apps can improve adherence to antimicrobial prescribing guidelines^18^ and this may be a particular advantage for improving the appropriateness of prescribing by doctors, who report often prescribing differently to guidelines, in contrast to nurse prescribers who report making decisions supported by local and national guidelines.^35^

This research represents the first multicentre study interviewing a representative sample of experienced and trainee doctors as well as non-medical prescribers using the MicroGuide app in their clinical practice. Strengths of the methodology include the fact that the interview topic guide was informed by a model of behaviour developed from a validated and comprehensive framework of behaviour change theories to prompt participants to consider a wide variety of potential influences of the app on their prescribing practice.

This study has a number of limitations. It is possible that the participant sample included a self-selected group of app enthusiasts, biased in favour of the app. Nonetheless, interviewees reported a number of shortcomings of the app, sufficient to constitute a major theme within the interview data. This study is part of a programme of research that includes a quantitative assessment of the impact of the MicroGuide app on prescribing behaviour and clinical outcomes, but prescribing data were not collected for the current study participants, so corroboration of self-reported prescribing behaviours was not possible. However, experience at the University of Iowa Hospitals and Clinics provides confidence that the value of MicroGuide to clinicians is sustained beyond the initial launch, with app content access events increasing steadily over a 14 month period.^23^ Finally, this research is limited to user experiences of just one smartphone app and findings may not be transferable to other antimicrobial prescribing apps, although preliminary data suggest a similar experience with other smartphone apps for infectious diseases and for acute oncology.^18^^,^^36^^,^^37^

The recognized culture of non-interference with antimicrobial prescribing decisions made by senior doctors^38^^,^^39^ underscores the importance of *getting it right first time*, particularly when research suggests that the majority of empirical prescriptions remain unchanged.^40^ It is noteworthy from interview testimony reported here that senior doctors were comfortable with delegating the antimicrobial choice decision to trainee doctors guided by the MicroGuide app. This may reflect increasing sub-specialization in medicine and/or a greater recognition of the value of infection specialists and the need for antimicrobial stewardship. Providing authoritative guidance in a concise and accessible format such as a smartphone app at the point of prescribing may prove instrumental to successful antimicrobial stewardship in hospitals, particularly in low- and middle-income countries, where specialist clinical microbiology or pharmacy resources may not be available.^25^

The extensive uptake of the MicroGuide app in UK hospitals is consistent with interview findings reported here of the perceived value of the app to users, but the impact of the app (if any) on outcomes including antimicrobial prescribing, resistance and clinical outcomes has not been evaluated and comprises the next focus of our research. Decision-support functionality has recently been developed for the MicroGuide app, to convert local treatment algorithms into a more user-friendly format. A randomized-controlled trial of this decision-support functionality is planned, to understand whether decision-support offers any added benefits in terms of clinical outcomes and antimicrobial stewardship.

Hospital doctors and nurse prescribers believed that MicroGuide expedited their work and reduced the time taken to initiate treatment. They felt it enhanced their confidence when on-call or working outside their area of expertise, increased their autonomy and reduced the number of calls to microbiology colleagues. Overall, they felt that the app improved their prescribing, enhanced their adherence to hospital guidelines and empowered them to question the decisions of more experienced staff. Whilst some believed that the availability of guidelines had the potential to ‘dumb down’ clinicians’ ability to think through problems independently, many felt that the additional information provided in MicroGuide improved their knowledge and constituted a convenient teaching tool.

## Supplementary Material

dlab111_Supplementary_Data
