# Supplementary material for: ‘It makes life so much easier’—experiences of users of the MicroGuide™ smartphone app for improving antibiotic prescribing behaviour in UK hospitals: an interview study
Source: JAC Antimicrob Resist. 2021 Aug 12;3(3):dlab111. doi: 10.1093/jacamr/dlab111 (PMC8496772; doi:10.1093/jacamr/dlab111)
Supplement: dlab111_Supplementary_Data [file dlab111_supplementary_data.docx]

**Supplementary data**

**Table S1.** MicroGuide Study Interview Topic Guide

| **COM-B** | **TDF** | **Interview Question (some questions are relevant to non-users)** |
| --- | --- | --- |
| Capability (psychological) | Knowledge | 1. How did you become aware of the MicroGuide App? 2. What is your understanding of the purpose of the App? |
| Capability (psychological) | Memory, Attention & Decision Processes | 1. Would you say that accessing MicroGuide is something you usually do? 2. How often would you say that you access MicroGuide (e.g. per day or per week)? 3. Do you ever forget to use the App? What do you use instead? 4. What efforts have you noticed to promote MicroGuide in your hospital? 5. What would help people to remember to use MicroGuide? |
| Capability (psychological) | Behavioural regulation | 1. Does anyone in the hospital monitor whether antibiotic guidelines are being followed? If yes, how? 2. Is feedback provided? How? 3. Do you think it would be helpful to have additional monitoring and feedback systems? 4. How should they work? |
| Capability (physical) | Skills | 1. In what ways do you access MicroGuide (e.g. Smartphone / Web viewer)? 2. How does MicroGuide help with your day-to-day clinical practice? 3. Is any training provided by your hospital? 4. Is any further training required for App users? |
| Capability (physical) | Beliefs about capabilities | 1. How difficult or easy do you think it is to access MicroGuide (App or web viewer)? 2. How difficult or easy do you think it is to use MicroGuide? 3. How could the App design be improved to make it easier (more intuitive) to use? |
| Opportunity (physical) | Environmental context and resources | 1. How would you describe the hospital culture when it comes to using MicroGuide? 2. What would you say are the barriers to using MicroGuide? What are the facilitators? 3. Does anything in the ward environment affect whether MicroGuide is used? (e.g. resources) |
| Opportunity (social) | Social influences | 1. Are you more or less likely to use MicroGuide when others are around or when you are alone? 2. Do you think the presence of other people affects use of MicroGuide? If so, how? 3. Are there opinion leaders in the clinical environment that influence others or lead by example? If so, who are they? 4. Do opinion leaders affect use of MicroGuide? If so, how? 5. What do you think could be done to change this for the better (if applicable)? |
| Motivation (reflective) | Social / professional role / identity | 1. Do you see yourself as someone who promotes use of MicroGuide to others? If so, how? If not, why not? 2. Do you think MicroGuide affects a prescriber’s autonomy (independence)? If so, how? 3. Do you think use of MicroGuide is affected by a prescriber’s grade / seniority? If so, how? 4. Have you ever done something to discourage others from using MicroGuide? If yes, why? 5. How much influence do you feel you have on the content or design of MicroGuide? |
| Motivation (reflective) | Beliefs about consequences | 1. Do you think that using MicroGuide influences your prescribing? If yes, how? 2. Does MicroGuide influence your working relationship with colleagues? If yes, how? 3. What do you think are any benefits of MicroGuide:    1. to the patient    2. to the doctor or prescriber    3. to the hospital    4. to the wider community? 4. Do you think there might be any negative consequences to using MicroGuide? If so, what are they? For whom? Could this be changed? How? |
| Motivation (reflective) | Intentions | 1. Have you made a conscious decision to use or continue to use MicroGuide? 2. How much effort does it take to incorporate MicroGuide into your day-to-day workflow? (Scale of 1-7 with 7 being most effort) |
| Motivation (reflective) | Goals | 1. What do you think are the goals that hospital management expects MicroGuide to deliver? 2. To what extent is following MicroGuide a priority for you personally? 3. How do you think the hospital could encourage clinicians to use MicroGuide and follow guidelines? |
| Motivation (automatic) | Reinforcement | 1. Are there any incentives in your hospital to accessing and following MicroGuide? 2. Are there any disincentives to accessing and following MicroGuide? |
| Motivation (automatic) | Emotion | 1. How do you feel if you prescribe an antibiotic without following MicroGuide? |
| Motivation (automatic) | Optimism | 1. How likely, in your view, is MicroGuide to be successful in the future? 2. How do you think the way you use MicroGuide might change as you become more senior? 3. How might MicroGuide be improved to make it more valuable to clinicians? |
| **REFERENCES**  Cane J, O'Connor D, Michie S. Validation of the theoretical domains framework for use in behaviour change and implementation research. *Implement Sci*. 2012 Apr 24;7:37. doi: 10.1186/1748-5908-7-37. PubMed PMID: 22530986  Michie S, Atkins L and West R. The Behaviour Change Wheel – A Guide to Designing Interventions. 1st Edition, 2014. Silverback Publishing. ISBN: 978-1-291-84605-8 ([www.behaviourchangewheel.com](http://www.behaviourchangewheel.com))  Steinmo SH, Michie S, Fuller C, Stanley S, Stapleton C, Stone SP. Bridging the gap between pragmatic intervention design and theory: using behavioural science tools to modify an existing quality improvement programme to implement "Sepsis Six". *Implement Sci*. 2016 Feb 3;11:14. | | |
